# Intestinal Anti-Inflammatory Activity of the Aqueous Extract from *Ipomoea asarifolia* in DNBS-Induced Colitis in Rats

**DOI:** 10.3390/ijms19124016

**Published:** 2018-12-12

**Authors:** Valéria Costa da Silva, Aurigena Antunes de Araújo, Daline Fernandes de Souza Araújo, Maíra Conceição Jerônimo Souza Lima, Roseane Carvalho Vasconcelos, Raimundo Fernandes de Araújo Júnior, Silvana Maria Zucolotto Langasnner, Matheus de Freitas Fernandes Pedrosa, Caroline Addison Carvalho Xavier de Medeiros, Gerlane Coelho Bernardo Guerra

**Affiliations:** 1 Department of Biophysics and Pharmacology, Biosciences Center, Federal University of Rio Grande do Norte, 59072-970 Natal, Brazil; vcs.biomed@gmail.com (V.C.d.S.); auriprinino@gmail.com (A.A.d.A.); roseane2202@gmail.com (R.C.V.); carolaufrn@gmail.com (C.A.C.X.d.M.); 2 Faculty of Health Sciences of Trairi, Federal University of Rio Grande do Norte, 59200-000 Santa Cruz, Brazil; daline_araujo@yahoo.com.br; 3 Laboratory of Pharmaceutical Technology and Biotechnology, Department of Pharmacy, Federal University of Rio Grande do Norte, 59012-570 Natal, Brazil; mairalima4@hotmail.com (M.C.J.S.L.); mffpedrosa@gmail.com (M.d.F.F.P.); 4 Department of Morphology, Histology and Basic Pathology, Biosciences Center, Federal University of Rio Grande do Norte, 59072-970 Natal, Brazil; araujojr.morfologia@gmail.com; 5 Research Group on Bioactive Natural Products, Department of Pharmacy, Federal University of Rio Grande do Norte, 59012-570 Natal, Brazil; szucolotto@hotmail.com

**Keywords:** intestinal inflammation, colitis, DNBS, rats, *Ipomoea asarifolia*, Convolvulaceae.

## Abstract

Inflammatory bowel disease is triggered by an uncontrolled immune response associated with genetic, environmental, and intestinal microbiota imbalance. *Ipomoea asarifolia* (IA), popularly known as “salsa” or “brave salsa”, belongs to the Convolvulaceae family. The aim of this approach was to study the preventive effect of IA aqueous extract in 2,4-dinitrobenzene sulfonic acid (DNBS)-induced colitis in rats. Rats pretreated with IA extract or sulfasalazine (SSZ) received intracolonic instillation of DNBS in 50% ethanol (*v*/*v*). IA extract presented a protective effect against intestinal inflammation, with improvement in the disease activity index and macroscopic damage. IA or SSZ significantly reduced myeloperoxidase activity, and also down-regulation of the gene expression of JNK1, NF-κβ-p65, STAT3, and decreased levels of TNFα, IL-1β, and increased IL-10, associated with a significant improvement of oxidative stress, in addition to a reduction in MDA and an increase of glutathione in colonic tissue. The protective effect of the extract was also confirmed in histological evaluation, showing preservation of the colonic cytoarchitecture. Immunohistochemical analysis revealed down-regulation of NF-κβ-p65, iNOS, IL-17, and up-regulation of SOCs-1 and MUC-2. IA extract presents antioxidant and anti-inflammatory intestinal properties, and proved to be a potential application for preventing damage induced by DNBS.

## 1. Introduction

Inflammatory bowel disease (IBD) is a chronic inflammatory disease that presents two major phenotypes: Crohn’s disease and ulcerative colitis [1,2]. These are recurrent diseases, presenting an inappropriate and continuous inflammatory response in a genetically susceptible individual. Current understanding of the pathogenesis of IBD provides insight into the relevant mechanisms of mucosal immunity, including how genetic factors interact with microbial and environmental antigens, as well as the biological control points involved in the mucosal immune response in a manner that results in different phenotypes of the disease [1,3].

The pathogenesis of intestinal inflammation is associated with genetic alterations and a defect or increase in mucosal sensitivity to intestinal lumen antigens, which are introduced into the naïve T cells for differentiation to take place in Th-specific cells (Th1, Th2 and Th17) after undergoing cell digestion by the antigen-presenting cells. The various components of the immune system involved in the pathogenesis of IBD includes intestinal epithelium, lymphoid cells, monocytes/macrophages, neutrophils, dendritic cells, T cells, and B cells, and their secreted mediators, such as cytokines and chemokines [4], especially the IL-1 cytokine family, tumor necrosis factor, IL-12, transforming growth factor-β, and IL-10 [5] which are responsible for regulating various aspects of the inflammatory bowel process, where the imbalance between inflammatory and anti-inflammatory mediators leads to a disturbance of the cellular microenvironment, contributing to the progression of tissue damage during inflammation [6].

*Ipomoea asarifolia* (Desc.) Roem. & Schult belongs to the Convolvulaceae family, popularly known as “salva or brava salva” in the Brazilian Northeast, being native and non-edemic in Brazil, occurring in the north, northeast, central-west, and southeast regions. It is predominantly present in the Amazon region, Atlantic forest, Caatinga, and Restinga in Brazil [7]. Despite *I. asarifolia* being considered toxic for cattle, sheep and goats [8,9], a recent study by Akindele et al. (2015) [10], showed that the hydroethanolic macerate of this plant when administered via the intraperitoneal (ip) route, is relatively safe up to a dose of 40–1000 mg/kg in a study of subchorionic toxicity (90 days) in rats. In popular medicine, a decoction of salsa leaves is used for various purposes, including the treatment of dermatitis, scabies, syphilis, skin ulcers, and external wounds [11]. Two studies have investigated *I. asarifolia* for its anti-inflammatory properties, one using a methanolic extract of the leaves (ip) in carrageenan-induced paw edema [12], and another using an aqueous maceration of aerial parts in an albumin-induced paw edema model [13]; both presented a preliminary phytochemical test and exercised significant antiedematogenic activity. In addition, pharmacological approaches have revealed anti-inflammatory properties of the aqueous extract and fractions of dichloromethane, ethyl acetate, and butanol (intravenous-iv) against carrageenan-induced inflammation of *Tityus serrulatus* venom in the peritonitis model [14], and also demonstrated anti-inflammatory potential in ear edema, peritonitis, and airbag inflammation models [15].

Therefore, the objective of the present study was to evaluate the preventive anti-inflammatory effects of *Ipomoea asarifolia* (IA) aqueous extract in the experimental model of rat colitis induced by 2,4-dinitrobenzene sulfonic acid (DNBS), a well-established model of intestinal inflammation that presents some histological alterations and biochemical characteristics of human disease [16], and to compare its effects with sulfasalazine (SSZ), an aminosalicylate currently used in the treatment of IBD.

## 2. Results

### 2.1. Effect of *Ipomoea asarifolia* Aqueous Extract on the Disease Activity Index, Macroscopic Score, and Weight/Colonic Length Relationship

The administration of IA extract at different doses (25, 50, and 100 mg/kg) over the three days before the induction of intestinal inflammation had no negative impact on body weight, feeding, or water consumption when compared to the healthy group. There was also no ptosis, diarrhea, piloerection, salivation, convulsions, somnolence, tremors, palpebral ptosis, and no mortality occurred. Still, we did not observe significant differences in glycemic levels, total cholesterol, and triglycerides (Table S1).

**Figure 1:**
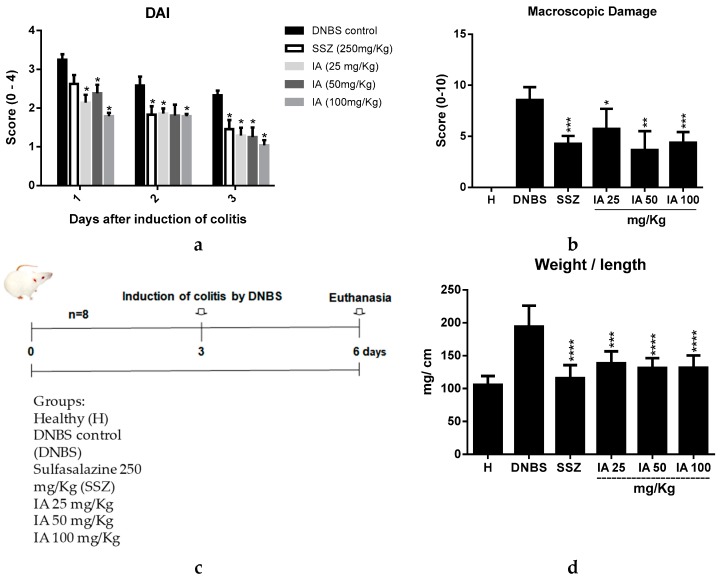
Experimental design and effect of 25, 50, and 100 mg/kg doses of *I. asarifolia* (IA) aqueous extract and 250 mg/kg of sulfasalazine (SSZ) on disease activity index (DAI) (**a**); macroscopic damage index (MDI) (**b**); experimental design (**c**); weight/length relationship of the colon (**d**) of the different groups in the experimental trial of acute colitis induced by 2,4-dinitrobenzene sulphonic acid (DNBS) in rats. H, Healthy group; DNBS, DNBS group; SSZ, Sulfasalazine group; and the 25, 50, and 100 mg/kg doses of IA. Data are expressed as mean ± SEM, (*n* = 8). Statistical differences * *p* < 0.05, ** *p* < 0.01, *** *p* < 0.001 and **** *p* < 0.0001 of treatments vs. DNBS control were presented.

Intracolonic administration of 30 mg of DNBS caused an intestinal inflammatory process availed by disease activity index (DAI) [17], clinically characterized by the presence of diarrhea, blood in the perianal region, and significant weight loss. Pre-treatment with IA 25, 50, and 100 mg/kg or SSZ 250 mg/kg promoted a significant improvement in the DAI values when compared to the DNBS control (*p* < 0.05) (Figure 1a).

**Figure 2:**
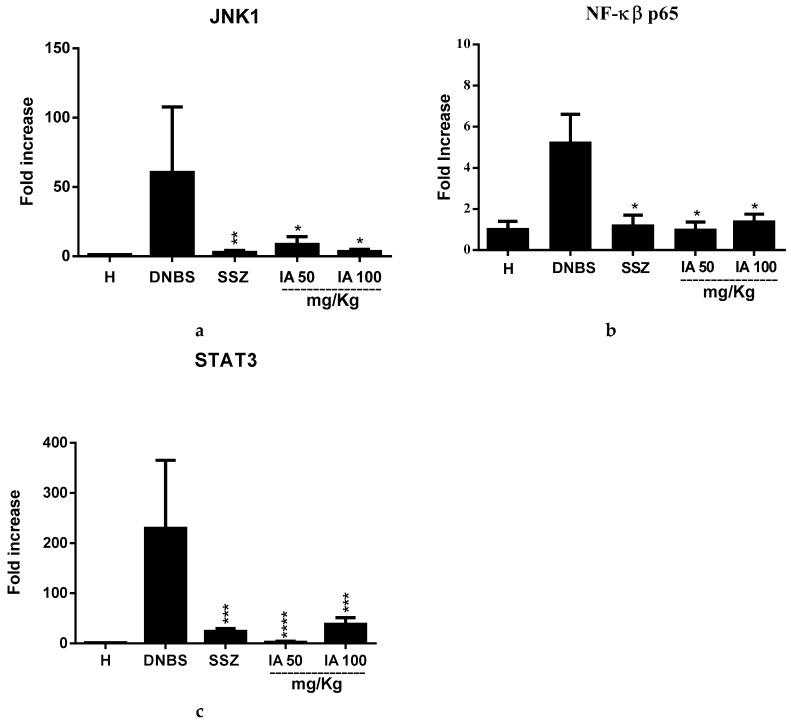
Effect of 50 and 100 mg/kg doses of *I. asarifolia* (IA) aqueous extract and 250 mg/kg sulfasalazine (SSZ) on the gene expression of c-Jun N-terminal kinases 1 (JNK1) (**a**), Nuclear factor kappa β (NK-κβ-p65) (**b**) and Activator of transcription 3 (STAT3) (**c**) in colonic tissue of the experimental trial of acute colitis induced by 2,4-dinitrobenzene sulfonic acid (DNBS) in rats. H, Healthy group; DNBS, DNBS group; SSZ, sulfasalazine group; and 50 and 100 mg/kg doses of the IA group. Data are expressed as mean ± SEM, (*n* = 8). Statistical differences * *p* < 0.05, ** *p* < 0.01, *** *p* < 0.001 and **** *p* < 0.0001 of treatments vs. DNBS control were presented.

The inflammatory bowel process induced by the intracolonic administration of DNBS was evaluated for its severity and the extent of lesions by applying the macroscopic damage index (MDI) [18]. The colonic damage observed in the DNBS control group was characterized by large areas of tissue damage, with the presence of ulcerations and intestinal wall thickening, leading to a mean MDI score of 8.5. Administration of SSZ and IA at all tested doses resulted in a significant reduction of this score, characterized by isolated sites of inflammation and/or ulceration, obtaining a mean MDI of 5.5, 3.5, 4.0, and 4.0 (*p* < 0.05) corresponding to a treatment with the IA extract at doses of 25, 50, and 100 mg/kg and SSZ (Figure 2b), respectively.

In addition, intestinal inflammation caused by DNBS induces shortening and thickening of the colonic tissue walls, therefore this was another parameter analyzed for characterization and differentiation of the inflammatory process [19,20,21] in the treated groups compared to the DNBS control. Beneficial effects of IA extract on the inflammatory process were observed, as it reduced the weight/length in all tested doses and SSZ (Figure 1d), thus corroborating the DAI and MDI data, revealing an improvement in the inflammatory process.

### 2.2. Effect of the *Ipomoea asarifolia* Aqueous Extract on the Gene Expresseion of JNK1, NK-κβ-p65, and STAT3 

Intra-colonic DNBS significantly up-regulated the gene expression of N-terminal kinases c-Jun 1 (JNK1), nuclear factor kappa β (NF-κβ-p65), and activator of transcription 3 (STAT3). However, pretreatment with 50 and 100 mg/kg of IA extract significantly down-regulated the expression of these markers from intestinal inflammation signaling pathways compared to the DNBS control (*p* < 0.05) and it was similar to treatment with SSZ.

### 2.3. Effect of the *Ipomoea asarifolia* Aqueous Extract on the Colonic Levels of TNF-α, IL-1β, and IL-10

**Figure 3:**
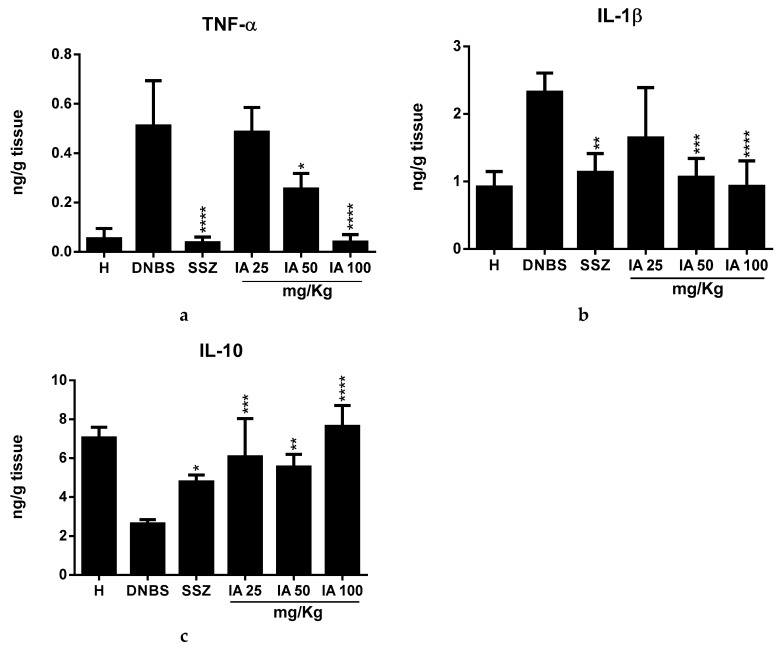
Effect of 25, 50, and 100 mg/kg doses of *I. asarifolia* (IA) aqueous extract, and 250 mg/kg sulfasalazine (SSZ) on the levels of cytokines, tumor necrosis factor α (TNF-α) (**a**); interleukin-1β (IL-1β) (**b**), and interleukin-IL10 (IL-10) (**c**) in the experimental trial of acute colitis induced by 2,4-dinitrobenzene sulfonic acid (DNBS) in rats. H, Healthy group; DNBS, DNBS group; SSZ, Sulfasalazine group; and 25, 50, and 100 mg/kg doses of IA. Data are expressed as mean ± SEM, (*n* = 8). Statistical differences * *p* < 0.05, ** *p* < 0.01, *** *p* < 0.001 and **** *p* < 0.0001 of treatments vs DNBS control were presented.

Colonic inflammation significantly increased tumor necrosis factor α (TNF-α) and interleukin-1β (IL-1β) levels, and reduced interleukin-10 (IL-10). Pre-treatment revealed that 50 and 100 mg/kg doses of IA extract significantly reduced TNF-α levels, with the 100 mg/kg dose being particularly similar to SSZ (Figure 3a) compared with the DNBS control group (*p* < 0.0001). IA at doses of 50 and 100 mg/kg and SSZ (Figure 3b) also significantly reduced the colonic levels of IL-1β (*p* < 0.05), while IL-10 levels significantly increased for all IA or SSZ doses (*p* < 0.05) compared to the DNBS control (Figure 3c).

### 2.4. Effect of *Ipomoea asarifolia* Aqueous Extract on Colonic Oxidative Stress

**Figure 4:**
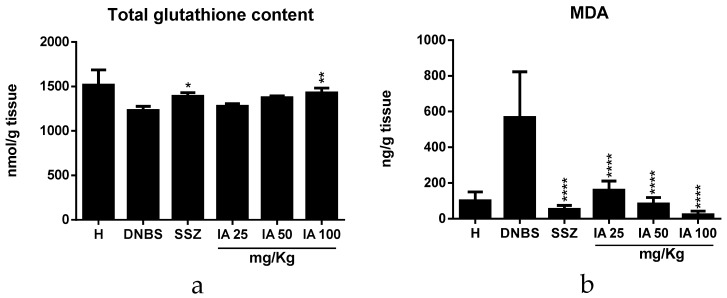
Effect of *I. asarifolia* (IA) aqueous extract at doses of 25, 50, and 100 mg/kg and sulfasalazine (SSZ) at 250 mg/kg on colonic oxidative stress by total glutathione content (**a**) and malondialdehyde (MDA) levels (**b**) in the experimental trial of acute colitis induced by 2,4-dinitrobenzene sulphonic acid (DNBS) in rats. H, Healthy group; DNBS, DNBS group; SSZ, Sulfasalazine group; and 25, 50, and 100 mg/kg doses of IA. Data are expressed as mean ± SEM, (*n* = 8). Statistical differences * *p* < 0.05, ** *p* < 0.01, *** *p* < 0.001 and **** *p* < 0.0001 of treatments vs DNBS control were presented.

Therefore, the IA extract at the 100 mg/kg dose increased total glutathione content (*p* < 0.01) (Figure 4a) and decreased malonildialdehyde (MDA) levels at all tested doses and SSZ (*p* < 0.0001) (Figure 4b) compared to the DNBS control; thus, the IA extract shows antioxidant potential.

### 2.5. Histological Analysis and Myeloperoxidase Activity

**Figure 5:**
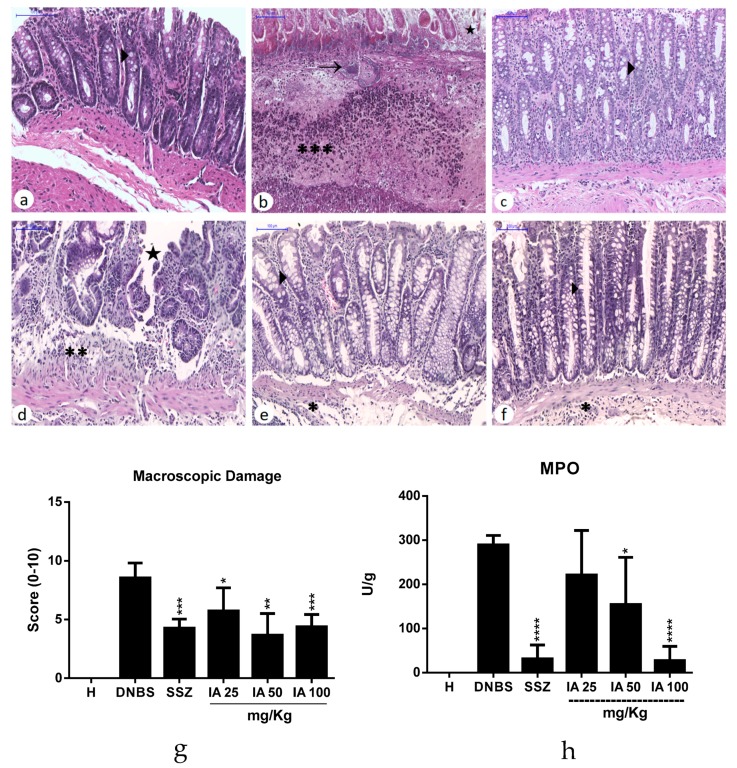
Histopathological analysis of the effect of the *I. asarifolia* (IA) aqueous extract at doses of 25, 50, and 100 mg/kg, and sulfasalazine (SSZ) at 250 mg/kg on intestinal colon mucosa in the experimental trial of acute colitis induced by 2,4-dinitrobenzene sulphonic acid (DNBS) in rats. Healthy group (**a**), exhibiting normal colonic mucosa with the presence of intact epithelium and numerous goblet cells; DNBS group (DNBS) (**b**), demonstrating the loss of epithelial integrity, intense inflammatory infiltrate (∗∗∗), focal necrosis (→) and ulceration (★); Sulfasalazine group (**c**), with characteristics similar to the saline group; IA dose of 25 mg/kg (**d**) presented areas with loss of epithelial integrity and moderate inflammatory infiltrate (∗∗); IA dose of 50 mg/kg (**e**) and IA dose of 100 mg/kg (**f**) exhibited better organized colonic epithelial tissue, similar to the saline group, and presented areas with loss inflammatory infiltrate (∗). The arrow heads (**►**) represent the goblet cells. Microscopic score of treatments vs. DNBS control (**g**). Myeloperoxidase activity (MPO) (**h**). H, Healthy group; DNBS, DNBS group; SSZ, Sulfasalazine group; and 25, 50, and 100 mg/kg doses of IA. Data are expressed as mean ± SEM, (*n* = 5). Statistical differences * *p* < 0.05, ** *p* < 0.01, *** *p* < 0.001 and **** *p* < 0.0001 of treatments vs DNBS control were presented (Scanner Panoramic Viewer, 100×).

Through the histological analysis of the colon of the healthy group (Figure 5a), it is possible to observe the preserved intestinal crypts and the presence of numerous goblet cells. The fibrous-type conjunctiva is interspersed with numerous usual fibroblasts. The DNBS control group (Figure 5b) shows the presence of a large colonic inflammation area characterized by ulceration and necrosis of epithelial cells. This colonic damage was associated with intense transmural inflammation, with infiltration of granulocytes in the lamina propria, submucosa, muscular, and serous layer. It was possible to observe that DNBS administration caused tissue damage involving mucosa and submucosa, characterized by atrophy and the degeneration of intestinal crypts, edema, and a reduction of goblet cells, meaning a loss of tissue cytoarchitecture, and severe leukocyte infiltration.

However, treatments with IA extract, especially at the doses of 50 and 100 mg/kg (Figure 5e–f), improved the preservation of intestinal tissue, and it can also be observed that the mucosa architecture appeared to be preserved with the presence of goblet cells with their preserved mucin content. In addition, a reduction in inflammation and infiltrate was also observed, being considered to be mild to moderate with a focal distribution. Microscopic score values (Figure 5g) were significantly reduced in these treatment groups (*p* < 0.05) compared to the DNBS control group.

The SSZ-treated group (Figure 5c) also showed a significant recovery of colonic lesions induced by DNBS. Similarly, pre-treatment with the IA extract was associated with a significant recovery of intestinal histology (*p* < 0.05) compared to the DNBS control group. This improvement was evidenced by a significant reduction in the ulcerated epithelial area.

As expected, the colonic myeloperoxidase activity (MPO) increased in the DNBS control when compared to the healthy group. The pre-treatment with IA 50 and 100 mg/kg and SSZ exhibited a significant reduction in intestinal myeloperoxidase levels (*p* < 0.05) (Figure 5h) compared to the DNBS control, thus reflecting a decrease in inflammatory infiltrate and an improvement in the intestinal inflammatory process in these groups.

### 2.6. Immunohistochemical Analysis

**Figure 6:**
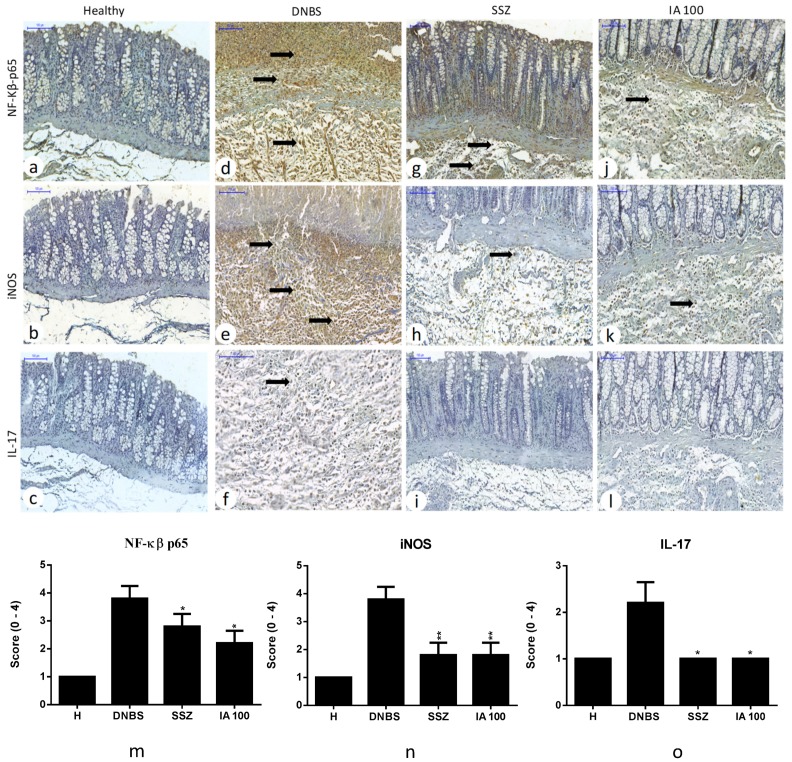
Immunohistochemical analysis of the effect of 25, 50, and 100 mg/kg doses of *I. asarifolia* (IA) aqueous extract and 250 mg/kg of sulfasalazine (SSZ) on intestinal colon mucosa in the experimental trial of acute colitis induced by 2,4-dinitrobenzene sulphonic acid (DNBS) in rats. Immunoreactivity was recognized by brownish staining in inflammatory cells, regardless of whether it was in the nucleus or cytoplasm. Healthy group without immunostaining for nuclear factor kappa β p65 (NF-κβ-p65), inducible nitric oxide synthase (iNOS), and interleukin-17 (IL-17) (**a**–**c**). DNBS group (**d**–**f**) exhibiting intense immunoreactivity for NF-κβ-p65 and iNOS in mucosa and submucosa, and discreet for IL-17. SSZ group (**g**–**i**) with moderate immunoreactivity for NF-κβ-p65 and discreet for iNOS and without immunostaining for IL-17. Group IA 100 mg/kg (**j**–**l**) showed discreet immunolabeling for NF-κβ-p65 and iNOS and without immunostaining for IL-17. Inflammatory cells (➡). Immunoreactivity score (**m**–**o**). H, Healthy group; DNBS, DNBS group; SSZ, Sulfasalazine group; and 25, 50 and 100 mg/kg doses of IA. Expressed as mean ± SEM, (*n* = 5). The statistical differences * *p* < 0.05 and ** *p* < 0.01 of treatments vs DNBS control are presented (Scanner Panoramic Viewer, 100×).

**Figure 7:**
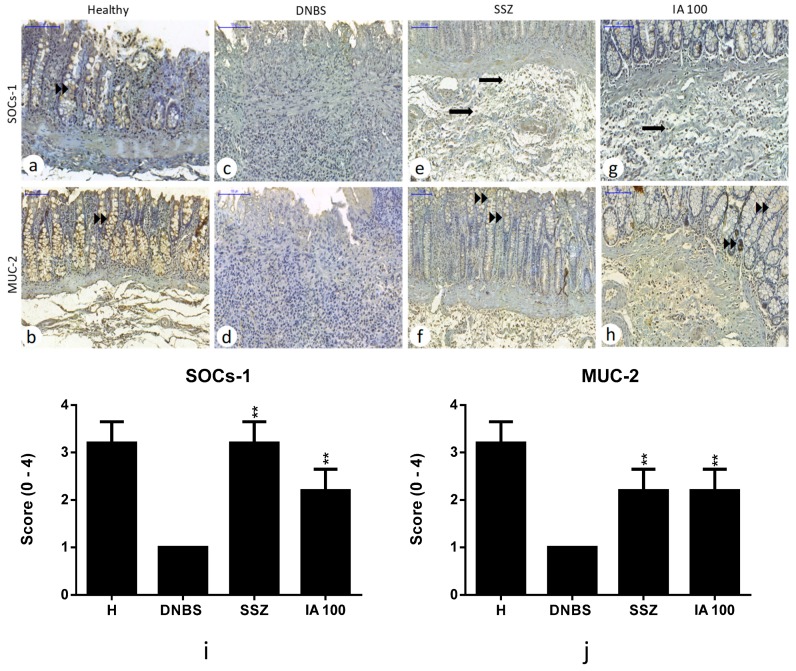
Immunohistochemical analysis of the effect of 25, 50, and 100 mg/kg doses of *I. asarifolia* (IA) aqueous extract, and 250 mg/kg of sulfasalazine (SSZ) on intestinal colon mucosa in an experimental trial of acute colitis induced by 2,4-dinitrobenzene sulphonic acid (DNBS) in rats. Immunoreactivity was recognized by brownish staining in inflammatory cells, regardless of whether in the nucleus or cytoplasm. Healthy group (**a**,**b**) exhibiting moderate immunoreactivity for cytokine-1 signaling suppressor (SOCs-1) and mucin-2 (MUC-2) in the epithelium (►►). DNBS control (**c,d**), exhibiting no immunoreactivity for SOCs-1 and MUC-2. SSZ group with moderate immunoreactivity for SOCs-1 and discreet for MUC-2 (**e,f**). Group IA 100 mg/kg with mild immunoreactivity for SOCs-1 and MUC-2 (**g,h**). Inflammatory cells (➡). Immunoreactivity score (**i,j**). H, Healthy group; DNBS, DNBS group; SSZ, Sulfasalizine group; and 25, 50, and 100 mg/kg doses of IA. Expressed as mean ± SEM, (*n* = 5). The statistical differences ** *p* < 0.01 of the treatments are presented vs DNBS control (Scanner Panoramic Viewer, 100×).

Immunohistochemical analysis revealed that pre-treatment with IA extract or SSZ significantly reduced the cell expression labeling for NF-κβ-p65 (*p* < 0.05), inducible nitric oxide synthase (iNOs) (*p* < 0.01), and interleukin-17 (IL-17) (*p* < 0.05) pro-inflammatory markers compared to the DNBS control. Also, the expression for cytokine-1 signaling suppressor (SOCs-1) and mucin 2 (MUC-2) intestinal barrier protein showed up-regulation (*p* < 0.01) compared to DNBS control, revealing tissue recovery after colitis induction by means of these treatments (Figure 6 and Figure 7).

## 3. Discussion

There are currently several therapeutic strategies for treating IBD, but they are associated with several adverse effects; therefore, there is an ongoing search for new therapies that may be beneficial in treating these chronic diseases [22]. In this sense, interest in the use of plant products for treating IBD has increased [23,24], especially those that are rich in phenolic compounds that present a wide spectrum of bioactivity, especially antioxidant activity, being able to act on different targets of the inflammatory process and protect the tissue microenvironment of oxidative stress.

DNBS induces an immune response of specialized Th1 cells, mainly involved in the pathogenesis of Crohn’s Disease [25]. Therefore, after inducing intestinal inflammation by DNBS, the parameters that can be analyzed include changes in body mass, clinical symptoms, changes in colonic cytoarchitecture, and analysis of changes in cytokine and MPO concentrations [1,16]. In addition, biochemical markers can be analyzed for characterizing the overall inflammation level, such as total glutathione content (GSH + GSSG), lipid peroxidation markers, and nitric oxide levels, as well as systemic inflammation indicators such as C-reactive protein and lipocalin-C [16].

Therefore, in this study, the anti-inflammatory effects of a plant species present in the semi-arid Brazilian northeast region that presents bioactive compounds such as caffeic acid, chlorogenic acid and rutin were evaluated, and it was described as presenting pharmacological properties such as antioxidant, anti-carcinogenic, and anti-inflammatory activities [26,27,28]. The high performance liquid chromatography (HPLC) analysis of IA extract enabled identifying cinnamic acid derivatives such as caffeic and clorogenic acids, as well as flavonoid rutin [14,15].

The results found in this study demonstrate the preventive effect of IA in the acute colitis model induced by DNBS. Administration of the extract or of SSZ caused a marked reduction in macroscopic colonic damage, with a decrease in inflammation along the colonic tissue being evidenced in the histological study, along with reduced leukocyte infiltration, preservation of the intestinal architecture, and up-regulation in MUC-2 expression, which is defective in IBD, and which may lead to the deficient stimulation of defensins and favor an imbalance of the intestinal microbiota [29]. The intestinal anti-inflammatory activity of the IA extract, especially at 50 and 100 mg/kg doses, can be attributed to its rich phenolic acid and flavonoid content, such as chlorogenic acid, caffeic acid, and rutin, respectively. Phenolic and flavonoid acids are involved in various biological processes such as the protection of deoxyribonucleic acid against xenobiotic agents, reducing inflammation and cell proliferation. Phenolic compounds can delay or even block carcinogenesis by inhibiting cell signaling pathways such as NF-κβ, involved in the regulation of cell proliferation, differentiation, and inflammation [30].

According to previous reports, rutin, a flavonoid present in IA extract, has been shown to exert intestinal anti-inflammatory effects in experimental models in the CD4+ CD62L+ T cell transfer model of colitis [31]. Vezza et al. (2016) [32] reports several studies that show the positive impact of flavonoids on intestinal inflammation in experimental rodent colitis models, including models that chemically induce colitis, such as acetic acid, trinitrobenzenesulfonic acid (TNBS), and sodium dextran sulfate (DSS), in genetically modified animal models such as HLA-B27 mice, or knockout mice for IL-10, and in cell transfer models, which show similarities with IBD in humans.

Zhang et al. (2017) [33] carried out a study with C57BL animals where chlorogenic acid (1 mM) was co-administered with DSS, and it was possible to observe a reduction in clinical signs of IBD, such as weight loss, colony weight/length ratio, histological improvement, and suppression of interferon-γ, TNFα and interleukin-6 (IL-6), colonic infiltration of F4/80+ macrophages, CD3+ T cells, and CD177+ neutrophils via the inhibition of the NF-κβ active signaling pathway. Similarly, caffeic acid inhibits NF-κβ, leading to a reduced production of proinflammatory cytokines, as demonstrated by the peptidoglycan polysaccharide-induced colitis assay in rats [34].

Colonic inflammatory status is closely related to forming reactive compounds from activated neutrophils and phagocytes, and by generating oxidative stress; this may be a mechanism underlying the pathophysiology of IBD [35]. The attack of reactive oxygen species on polyunsaturated fatty acids from cellular phospholipid membranes can progress with the formation of peroxyl radicals, which attack the membrane and lead to the destruction of its structure. Secondary products are formed during this process, such as MDA, a dialdehyde that can be detected in biological samples and is widely used to assess oxidative stress [36,37]. Thus, decreased MDA levels at all tested doses of the IA extract, and an increase in total glutathione in the treatment group with 100 mg/kg of IA demonstrate its antioxidant potential.

Maintenance of the intestinal mucosal architecture is essential for inhibiting the inflammatory response in IBD. In this sense, a histological analysis of the colon of the animals submitted to intestinal inflammation with DNBS and pre-treated with IA extract revealed a decrease in the inflammatory infiltrate, being one of the explanations for maintaining the tissue architecture with the presence of goblet cells. In IBD, an intense infiltration of polymorphonuclear cells into the intestinal mucosa is associated with the disease severity, which generally leads to severe epithelial damage [38,39] characterized by persistent infiltration of neutrophils at the injured site [35]. Polymorphonuclear induced tissue injury is often associated with the release of a variety of inflammatory mediators [39]. This result is in agreement with those of MPO, resulting from a decrease in the activity of this enzyme in the treatment with the IA extract or SSZ.

During the colonic inflammatory process, the activated macrophages stimulate inflammation through secreting cytokines such as TNF-α and IL-1β, as well as expressing other mediators of the inflammatory process, such as inducible nitric oxide synthase (iNOS); this is predominantly expressed in the intestinal inflammation sites [40], which had their expression reduced in pre-treatments with SSZ, and with a dose of 100 mg/kg of IA when compared to the DNBS control.

The inhibitory effect of colonic mucosal cell infiltration and cytokine production exerted by both SSZ and IA was specifically corroborated by the inhibition of the gene expression of intestinal inflammation signaling pathways such as mitogen-activated protein kinases (MAPKs) and NF-κβ-p65, the molecular target for IBD [41,42]. The activation of MAPKs (p38, ERK and JNK) plays a critical role in the activation of NF-κβ-p65 pathway, which mediates intracellular signal transduction involving the expression of the proinflammatory cytokines IL-1β, IL-6, and TNF-α. Chlorogenic acid (chA), a phenolic compound found in the IA extract, demonstrated its activity in the inhibition of MAPK pathways, such as c-Jun N-terminal kinases 1 (JNK1) and signaling regulated kinases 1 (ERK1), and in the inhibition of the expression of the phosphorylated proteins p-JNK1 and p-ERK1 [43]. Similarly, STAT3-mediated activation of acquired immune responses plays a pathogenic role in colitis by enhancing T cells survival. TNF-α, IL-6, NF-κβ, and STAT3 turned out to be associated with intestinal inflammation, leading to a predisposition to colorectal cancer associated with colitis. Thus, inhibition of the NF-κβ and STAT3 factors appears to be associated with clinical benefits in IBD, where the inhibition of persistent activation of STAT3 by NF-κβ may be an alternative in explaining the pathogenesis and treatment of colorectal cancer associated with colitis [44].

In addition, the inhibition of inflammassoma, characterized by the activation of caspase 1, necessary for the proteolytic cleavage of cytokinins such as pro-IL1-β and pro-IL18, in their biologically active forms [45,46] by small molecules such as flavonoid rutin [46], may partly explain the anti-inflammatory activity that is exerted by the IA extract.

In this context, improvement in the altered immune response was observed to be exerted by IA, as evidenced by the reduced colonic levels of inflammatory cytokines. Another important result in controlling mucosal damage are the levels of IL-10, an anti-inflammatory cytokine that is mainly produced by regulatory T lymphocyte cells [47], which is capable of inhibiting the production of IL-1β, IL-6, and TNF-α, as well as other proinflammatory cytokines of monocytes and macrophages [48]. In addition, increased SOCs-1 expression, which is a negative regulator for cytokine-mediated signaling [49], suggests that the extract has positive regulation in anti-inflammatory systems.

Therefore, the increase in IL-10 reinforces the beneficial effects of the IA extract, since it promotes the maintenance of intestinal homeostasis during inflammation [50], and also induces anti-inflammatory effects in many leukocytes, thus having a protective effect during intestinal inflammation [51]. Therefore, with a down-regulation in the gene expression of JNK1, NF-κβ-p65, and STAT3 proinflammatory cytokines, TNF-α, IL-1β, and IL-17, the increase of IL-10, an anti-inflammatory cytokine by IA pre-treatment, reveals an ability to modulate cytokines in the inflamed colon, being one of the possible mechanisms of intestinal anti-inflammatory activity that is observed during the experimental study, and that is associated with up-regulation in SOCs-1 and MUC-2 expression.

## 4. Materials and Methods

### 4.1. Plant Material and Preparation of Extracts

The collection of *I. asarifolia* leaves occurred in the city of Mossoró/RN, Brazil (latitude: −5.1875; longitude: −37.344167). The voucher specimen (MOSS 7096) was deposited at the “Dárdamo Andrade Lima” herbarium of the Federal Rural University of the Semi-arid/UFERSA, Brazil. The collection was authorized by the System of Authorization and Information on Biodiversity (SISBIO), process number 35017, and process number A4E8DE0 of authorization from the National System for the Management of Genetic Heritage and Associated Traditional Knowledge. The leaves were oven-dried and decocted for 15 min, with the proportion of vegetable drug:solvent being 1:10 (*w*/*v*). The aqueous extract fraction was obtained by freezing for 72 h at −80 °C.

Our study group performed a phytochemical analysis of the aqueous extract from *I. asarifolia* leaves by thin layer chromatography (TLC), in which the presence of phenols, tannins, alkaloids, saponins, and flavonoids was detected [14]. The same extract was subsequently analyzed by high performance liquid chromatography coupled to a mass spectrometer (HPLC-DAD), in which 10 main peaks were observed at 340 nm. Three peaks were identified from these 10 peaks, namely peak 2 (*t*R = 8.3 min), peak 4 (*t*R = 13 min), and peak 5 (*t*R = 24 min), as chlorogenic acid, caffeic acid, and rutin respectively, and the retention time was compared with standards, UV spectra, and co-injection (standard + extract). The extract was analyzed by high affinity liquid chromatography coupled to a mass spectrometer (LC-DAD-MS) in the same study, in which it was possible to confirm the presence of these compounds by the *m*/*z* values of 353, 179, and 609 for chlorogenic acid, caffeic acid, and rutin, respectively [15].

### 4.2. Animals

The project was approved by the Animal Use Ethics Committee (CEUA) of the Biosciences Center of the Federal University of Rio Grande do Norte, under protocol no. 068 (18 December 2015), with the following determinations for maintaining the animals: circadian cycle of 12 h (light/dark), controlled ambient temperature of 22 ± 3 °C, and relative humidity between 50 and 55%. Free access was given to extruded feed and drinking water (ad libitum). *Rattus norvegicus*, lineage Wistar, females, (*n* = 48), 8 to 10 weeks of age, and mean weights of 200 ± 20 g were used.

### 4.3. Experimental Design

Rats were randomly divided into groups (*n* = 8): healthy (H); DNBS group; IA (25, 50, and 100 mg/kg) and SSZ. They were given saline (1 mL/kg), IA (25, 50, or 100 mg/kg/day), or sulfasalazine (250 mg/kg/day) orally for two days before inducing colitis, and 48 h after. All animals were euthanized 72 h after the instillation of DNBS, with ip. thiopental (100 mg/kg) (Figure 1c).

### 4.4. DNBS-Induced Colitis

On the third day of the pre-treatment, and following 12 hr of fasting, the animals were anesthetized with 10% ketamine (50 mg/kg) and 2% xylazine (5 mg/kg) for the intracolonic administration of DNBS 30 mg dissolved in 250 μL of ethanol (50% *v*/*v*) by the means of a Teflon catheter at a distance of 8 cm from the perianal region per animal, and they were kept upside down for a period of 15 min to prevent the DNBS solution from escaping [52]. The negative control received 0.9% saline solution application in the same environment.

### 4.5. Evaluation of Intestinal Inflammation 

The evolution of the inflammatory bowel process was performed by determining the disease activity index (DAI), adapted from Cooper et al. [17]. The animals were weighed daily and evaluated for the presence of diarrhea and blood in the perianal region and/or in the feces. At the end of the treatment, the animals were euthanized, and the colonic segment was removed and cleaned of fat and mesentery, weighed, and the lengths were measured. After being opened longitudinally, the macroscopic damage index was evaluated according to Bell et al. [18], with scores ranging from (0) normal colon (no damage); (2) ulceration without hyperemia or wall thickening; (3) ulceration with flash point; (4) two sites or more of ulceration and/or inflammation; (5) large inflammation zones and ulceration with an extension greater than 1 cm and (6–10) for large areas of tissue damage and for damage with an extension greater than 2 cm, with 1 point being added for each additional centimeter of tissue injury; finally, the length and weight of the colon were measured.

Colon tissue samples were separated longitudinally and frozen at −20 °C for determining biochemical and immunological parameters. The MPO activity essay, which constitutes a sensitive marker of neutrophil infiltration and inflammation, was performed using the method described by Krawisz et al. [53].

Determination of total glutathione (GSH) content, which is an indirect estimation of the oxidative damage produced by an inflammatory process, was determined by the method described by Anderson [54]. MDA level analysis, which is an end product of lipid peroxidation and an important marker of oxidative stress, were performed by the method described by Esterbauer et al. [55].

The cytokine levels in the colonic tissue were evaluated using the protocols of the kits (Sigma-Aldrich, São Paulo, Brazil), using standard capture and detection antibodies for IL-1β, TNF-α, and IL-10, where colony tissue specimens were weighed, homogenized with phosphate buffer (10 mM, pH 7.2–7.4), and centrifuged at 3500× *g* at 4 °C for 10 min so that the supernatant from the centrifugation was used to determine the cytokines at an absorbance of 450 nm in ELISA reader.

### 4.6. Gene Expression Analysis

The colonic tissue was sectioned into fragments for RNA extraction, stored in RNAse-free eppendorfs, and lyzed in TRIzol solution. All RNA samples were quantified with the NanoDrop™ spectrophotometer determined from the optical density at a wavelength of 260 nm (using an OD_260_ unit equivalent to 40 μg/mL RNA). Ten micrograms of isolated total RNA were reverse transcribed to complementary DNA (cDNA) in a reaction. The cDNA was stored at −80 °C until further use. Gene expression was evaluated by PCR amplification using primer pairs based on published rat sequences (β-actin—*Rattus norvegicus*: Forward primer: CGCACTGCCGCATCCTCT, Reverse Primer: GTCGAAGAGAGCCTCGG; JNK1—*Rattus norvegicus* mRNA Forward primer: TGCTGATCGCCAGATCTCAG, Reverse Primer: ACAAGCCAGGTGTGAACGC; NF-κβ-p65—*Rattus norvegicus* v-rel avian reticuloendotheliosis viral oncogene homolog A (Rela), Forward primer: TCTGCTTCCAGGTGACAGTG, Reverse Primer: ATCTTGAGCTCGGCAGTGTT and STAT3 *Rattus norvegicus*: Forward primer: GGAGGAGGCATCGGAAAGT, Reverse Primer: TACCTGGGTCAGCTTCAGGG.

Quantitative RT-PCR was performed using Power SYBR Green master mix (Applied Biosystems, USA), and a Step One Plus thermocycler (Applied Biosystems, Foster city, CA, USA), according to the manufacturer’s instructions. For the 1× PCR master mix, 2.0 μL of each cDNA was added in a final volume of 10 μL. The relative quantitative fold change compared with the Healthy control (H) was calculated using the comparative *C*t method, where *C*t is the cycle number at which fluorescence first exceeds the threshold. The *C*t values from each sample were obtained by subtracting the values for GADPH Ct from the target gene *C*t value. The specificities of the resulting PCR products were confirmed by melting curves.

### 4.7. Histopathological Analysis

For histopathological analysis (*n* = 5), colic tissue samples were removed and fixed in 10% buffered paraformaldehyde in phosphate buffer (pH 7.2) for a period of up to 48 h, followed by processing by commonly used histological techniques, including the cutting of colon sections by microtome (3–4 μm) for fixation onto microscope slides, followed by eosin/hematoxylin staining, which were evaluated by Scanner Panoramic Viewer software, applying scores from 0 to 3, as adapted from Wirtz et al. [56], for the absence of goblet cells, the presence of inflammatory cells, mucosal and serous cell infiltration, the destruction of tissue architecture, ulceration, and abscess in crypts.

### 4.8. Immunohistochemical Analysis

Sections of histological blocks 3 μm thick (*n* = 5) were made for fixation on silanized slides by dewaxing and rehydration techniques. The samples were washed with 0.3% Triton X-100 in phosphate buffer, treated with 3% hydrogen peroxide, and incubated at 4 °C overnight with the following primary antibodies and respective dilutions: NF-κβ-p65, 1:400; IL-17, 1:1000; iNOS, 1:500; SOCs-1, 1:800; and MUC-2, 1:500. The next day, the slides were washed, and the secondary antibody + biotin was added with phosphate buffer, and streptavidin–HRP (peroxidase enzyme) conjugated for 30 min. The slides were analyzed by Scanner Panoramic Viewer software. Immunoreactivity was recognized by brownish staining in inflammatory cells, regardless of whether it was in the nucleus or cytoplasm. Determination of the immunostaining intensity of the cells was determined by an adaptation of the protocol described by Guerra et al. [57], with scores of (1) without positive markers for labeling; (2) reduced number of positive cells or isolated cells (<10%); (3) moderate number of positive cells (11–50%), and (4) large number of (> 50%) positive cells.

### 4.9. Statistical Analysis

The results were expressed as mean ± SEM. Data were analyzed by one-way ANOVA, followed by Tukey’s post-test and Mann–Whitney methods for parametric and non-parametric data, respectively. GraphPad 6.0 software was used for this analysis, for determining significant differences (*p* < 0.05) between the samples.

## 5. Conclusions

In summary, our results show preclinical evidence that *Ipomoea asarifolia* aqueous extract exerts an anti-inflammatory intestinal preventive effect in DNBS-induced colitis in rats. This extract minimizes the clinical signs and modulates the inflammatory response by suppressing JNK1, NF-κβ-p65, and STAT3, and up-regulating SOCs-1, consequently reducing the colonic levels of TNF-α, IL-1β, and IL-17 proinflammatory cytokines. On the other hand, it increased levels of IL-10, an important anti-inflammatory cytokine. Also, a decrease in MPO, an enzyme released during neutrophil activation, was observed, as well as a decrease in MDA, a product of lipid peroxidation that restored total glutathione content and down-regulation in iNOS expression. In addition, the beneficial effect of the extract preserved the tissue cytoarchitecture, which is associated with an up-regulation in MUC-2. Finally, the data indicate that the IA has preventive effects in DNBS-induced intestinal inflammation in rats, revealing its potential in managing human IBD.

## Supplementary Materials

The following are available online at http://www.mdpi.com/1422-0067/19/12/4016/s1.

## Abbreviations

DAI	Disease activity index
DNBS	2,4-Dinitrobenzene sulfonic acid
IA	*Ipomoea asarifolia*
IBD	Inflammatory bowel disease
IL-10	Interleukin-10
IL-17	Interleukin-17
IL-1β	Interleukin-1β
iNOS	Inducible nitric oxide synthase
JNK1	c-Jun N-terminal kinases 1
MDA	Malonildialdehyde
MDI	Macroscopic damage index
MPO	Myeloperoxidase
MUC-2	Mucin-2
NF-κβ	Nuclear factor kappa β
SSZ	Sufasalazine
SOCs-1	Cytokine signaling suppressor-1
STAT3	Activator of transcription 3
TNF-α	Tumor necrosis factor-α
