# Supplementary material for: Intestinal Anti-Inflammatory Activity of the Aqueous Extract from Ipomoea asarifolia in DNBS-Induced Colitis in Rats
Source: Int J Mol Sci. 2018 Dec 12;19(12):4016. doi: 10.3390/ijms19124016 (PMC6321343; doi:10.3390/ijms19124016)
Supplement: Supplementary file 1 [file ijms-19-04016-s001.pdf]

SUPPLEMENTARY MATERIAL

TABLE 1. Effect of administration by gavage of the aqueous extract of *Ipomoea asarifolia* on glucose, triglycerides, total cholesterol, somnolence, tremors, convulsions, palpebral ptosis, piloerection, salivation, diarrhea, mortality and weight.

Groups	Glucose mg/dL	TG mg/dL	CT mg/dL	Somnolence	Tremors	Convulsion	Ptosis	Piloerection	Salivation	Diarrhea	Mortality-Number per group/death	Weith 1º day	Weith 3º day
H (saline)	107,80 ± 17,60	33,33 ± 4,80	60,00 ± 11,58	absence	absence	absence	absence	absence	absence	absence	8/0	192 ± 2,64	183,33 ± 1,52
IA 25	120,29 ± 41,65	48,50 ± 18,96	65,71 ± 10,36	absence	absence	absence	absence	absence	absence	absence	8/0	197,87 ± 20, 35	184,25 ± 18, 86
IA 50	80,17 ± 27,13	38,00 ± 11,03	77,33 ± 19,65	absence	absence	absence	absence	absence	absence	absence	8/0	194,12 ± 13,24	183,5 ± 9,76
IA 100	72,67 ± 25,16	43,83 ± 6,79	70,29 ± 8,32	absence	absence	absence	absence	absence	absence	absence	8/0	194,12 ± 21,44	191,5 ± 19,96

Values represent the mean ± SEM (n = 8/group). TC, total cholesterol; TG, Triglycerides. Healthy group, H; and 25, 50 and 100 mg/Kg doses of *I. asarifolia*, IA.
